# Editorial: Metal-complexed molecules of natural and synthetic origin: Pharmacological advances and therapeutic applications

**DOI:** 10.3389/fphar.2022.1044439

**Published:** 2022-10-06

**Authors:** Ranjan K. Mohapatra, Veronique Seidel, Mohammad Azam

**Affiliations:** ^1^ Department of Chemistry, Government College of Engineering, Keonjhar, Odisha, India; ^2^ Natural Products Research Laboratory, Strathclyde Institute of Pharmacy and Biomedical Sciences, University of Strathclyde, Glasgow, United Kingdom; ^3^ Department of Chemistry, College of Science, King Saud University, Riyadh, Saudi Arabia

**Keywords:** metal complexes, density functional theory, biological properties, molecular docking studies, SARS-CoV-2

In recent years, there has been a growing interest in metal-based (particularly coordination) compounds owing to their structural variations and potential to be exploited for diverse biological applications. Many diseases, including the recent COVID-19 pandemic - caused by consecutive waves of severe acute respiratory syndrome coronavirus 2 (SARS-CoV-2) variants - continue to have significant impact on healthcare systems worldwide. This Research Topic contains a collection of articles that include computational studies (particularly molecular docking to predict binding affinities and interactions of ligands to specific protein targets) and *in vitro* biological testing to evaluate the pharmacological potential of metal-complexed molecules.

Muhammad et al. presented the synthesis of four new carboxylate organotin (IV) complexes using a 4-chlorophenoxyacetate ligand. The structure of these compounds was confirmed by FT-IR, NMR, and single crystal XRD analysis. These compounds showed appreciable cytotoxic effects against lung cancer (A549) and normal lung fibroblast (MRC-5) cell lines. They also showed antibacterial activity, higher than that of the free ligand. A molecular docking study showed that these compounds had adequate interactions with the SARS-CoV-2 spike and nucleocapsid proteins, as well as with the human angiotensin converting enzyme (ACE_2_).

In another study, Kljun et al. evaluated the inhibitory activity of ten organoruthenium compounds with hydroxyquinolinate and diketonate ligands toward aldo-keto reductase 1C (AKR1C) enzymes. These compounds were potent inhibitors of the AKR enzymes. Their mechanism of action involved two inhibitor molecules binding to the enzyme in a first fast and reversible step and a second slower and irreversible step. Moreover, these compounds displayed anticancer potential toward the chemoresistant ovarian cancer cell line COV362 that was similar to cisplatin, but better than carboplatin.

Mohmad et al. reported on the optimal conditions for the formation of an iridium (III)-6-chloro-3-hydroxy-7-methyl-2-(2′-thienyl)-4-oxo-4*H-*1-benzopyran (CHMTB) complex and determined its electronic properties using a computational approach. This complex showed antibacterial and radical-scavenging activity.

In another study, Yadav et al. reported a detailed computational study on five α-aminophosphonate ligated mono and dinuclear Cu(II) complexes. The mononuclear species showed a higher binding affinity for the targets than the dinuclear species in a molecular docking study against two SARS-CoV-2 proteases.

Taken together, the papers collected in this Research Topic showcase the most recent methods used to characterize metal-complexed molecules, and predict/test for their pharmacological activity. We believe that this collection of articles highlights some promising therapeutic applications for metal-complexed molecules and hope it will be useful for all researchers working in this field.

